# DeepTrackSecure: an integrated classification-detection system with predictive risk analytics for proactive railway safety management

**DOI:** 10.3389/frai.2026.1800348

**Published:** 2026-05-15

**Authors:** P. Balakrishnan, A. Anny Leema, S. Suresh Nagarajan, K. S. Rashmika, J. Rahul Kumar

**Affiliations:** School of Computer Science and Engineering, Vellore Institute of Technology, Vellore, India

**Keywords:** ablation study, deep learning, edge and cloud deployment, image classification, object detection, railway track fault detection, random forest, ResNet50

## Abstract

Railway track failures such as cracks, displacements, corrosion, and missing fasteners pose serious safety risks and require timely detection and prioritization. Manual inspection techniques are time-consuming, prone to mistakes, and unsuitable for extensive monitoring. This paper suggests DeepTrackSecure, a multi-stage deep learning framework for railway track fault detection and severity-aware risk prediction, to overcome these constraints. The suggested system uses YOLOv5 for accurate fault region localization in defective images after using ResNet50 as an initial screening model to categorize railway track images as defective or non-defective. By combining defect area, detection confidence, and defect frequency, a quantitative severity score is created that goes beyond traditional detection and allows for well-informed risk assessment. Random Forest and XGBoost models are assessed for classifying track conditions into low, medium, and high-risk levels based on this severity representation, exhibiting dependable and consistent decision-making. Ablation experiments to compare YOLO-only detection, classification-detection fusion, and the full severity-based risk prediction model confirm the role of each of the system components. Also, runtime and memory analysis are used to ensure that the performance is near to what is required in edge and cloud deployment and that the robustness is tested in harsh conditions like blur, noise, and low-light conditions. The findings of the experiments indicate that DeepTrackSecure functions as a decision-support and maintenance prioritization tool that produces severity-derived risk proxies (Low/Medium/High) validated against expert opinion. The system is intended to assist maintenance scheduling decisions rather than serve as a directly deployable operational safety system; broader deployment should be preceded by recall improvement and validation against real failure-event ground truth.

## 1 Introduction

The issue of rail safety is paramount due to the necessity of railway transportation in the movement of people and goods worldwide. Structural defects in railway tracks that may lead to disastrous accidents such as derailments and significant crashes include cracks, misalignments, corrosion, and missing fasteners. Consequently, to ensure safe and reliable railway operations, it is important to detect and fix such defects in a timely manner either by checking them by hand or using special testing devices, which are the primary type of railway track inspection method. These are labor intensive, time consuming, costly, and highly dependent on human expertise, hence cannot be applied to large-scale and continuous monitoring despite being partially effective. Moreover, human inspections are susceptible to human error and most of the time fail to deliver timely risk assessment in dynamically evolving environmental conditions. The convolutional neural networks (CNNs) have been found to be very successful in detecting small imperfections in the image data and in learning complex visual patterns. Consequently, a number of studies have investigated the use of classification and object detection networks for image-based railway fault detection. Nevertheless, without offering a systematic evaluation of defect severity or operational risk, the majority of current methods concentrate mainly on locating or detecting defects.

Finding a flaw is not enough in real-world railway maintenance situations. Making maintenance decisions requires knowing how serious a defect is, how often it happens, and how quickly it needs to be fixed. Many detection-based systems currently in use have limited practical applicability due to the lack of severity-aware risk quantification. Furthermore, few studies offer robustness assessment in challenging circumstances like dim lighting, noise, and motion blur, or ablation-based validation to support multi-stage architectures. This paper suggests DeepTrackSecure, a multi-stage deep learning framework for predictive risk analytics and railway track fault detection, to overcome these drawbacks. The suggested system incorporates YOLOv5 for accurate fault localization after a quick defect screening stage using ResNet50. Beyond detection, defect size, detection confidence, and defect frequency are combined to create a quantitative severity score. Next, actionable risk levels—low, medium, or high are predicted using ensemble learning models based on this severity representation. The suggested framework is appropriate for implementation in edge and cloud-based railway monitoring systems since each system component’s efficacy is methodically verified through ablation studies, robustness testing in real-world scenarios, and computational efficiency analysis.

ResNet50 and YOLOv5 are well-known architectures, however, the main novelty of this paper is the maintenance-oriented formulation that links detection evidence to maintenance decisions. For this purpose, we developed a conditional two-stage pipeline that filters images before localization so that only necessary detections get attention, and we also created a novel defect severity representation (extent/area, confidence, and defect frequency) that is later converted into understandable risk levels (Low/Medium/High) for maintenance task prioritization. We demonstrate the helpfulness of each stage via ablation, robustness testing, and deployment-oriented runtime/memory evaluation. The core novelty of this work is not the use of ResNet50 or YOLOv5 individually, but the decision-theoretic pipeline that connects raw detection evidence to actionable maintenance decisions. Specifically: (i) Problem reformulation: We recast railway inspection as a maintenance prioritization task, whose output is an interpretable risk label (Low/Medium/High) directly usable by maintenance staff, rather than a detection task reporting bounding boxes or mAP. (ii) Evidence-based severity score: We present a principled, bounded severity score S which is a combination of defect area, detection certainty and defect frequency into one formula. None of the previous works reviewed are using such a combination of these three signals with limited properties and expert-calibrated thresholds. (iii) Conditional inference design: ResNet50 screening gate avoids unnecessary YOLOv5 calls on the images which are not defects and saves compute to deploy it to large scale, is an efficiency design that is not available with detection-only systems. Staged, multi-criteria validation (iv) We verify every pipeline stage separately: through ablation, testing adversarial robustness, and expert-agreement analysis, and offer a rigorous evidence base that extends past reporting a single final metric. Overall, the input is the entire inspection-to-decision pipeline architecture and its multi-level verification and not the model components.

Recent studies have explored two-stage pipelines that combine CNN-based screening with YOLO-based localization (Rodríguez-Abreo et al., 2024). However, our work is designed specifically for maintenance decision support, by linking localized defect evidence to (i) an evidence-driven severity representation and (ii) interpretable Low/Medium/High risk outputs for prioritization.

## 2 Literature review

Some more current examples have implemented YOLO-based detectors to inspect the faults of a railway infrastructure using image-based information that is recorded in a real-life scenario (Yilmazer and Karakose, 2023). To improve the quality of captured images and the precision of the detection process, the system combines video stabilization that relies on the OpenCV library (Nandan et al., 2023). The model has 250 epochs, and it is trained on a dataset of 500 images, with the assistance of the LabelImg. The dataset is applied to training and testing. The method can identify different forms of faults including rail edge breaks, missing fasteners, and sleeper cracks at mean average precision (mAP) of 94.5% and a recall of 97.3%. This contactless method can be applied to real-life railway monitoring as this is faster, safer and more versatile compared to conventional inspecting (Hashmi et al., 2022). The articles by Minguell and Pandit (2023) were devoted to the comparison of the YOLOv5, Faster R-CNN, and EfficientDet to detect faults on the railway tracks as an image set. The two articles mentioned the safety and security concerns of trains with the application of machine learning. One article mentioned how to predict rail incident risks through a data-driven method, whereas the other is a YOLOv5 that is enhanced to detect and prevent illegal individuals who proceed to the track due to the security breaches foundation (Hadj-Mabrouk, 2020; Yang and Palaoag, 2024). The study of another author suggests a multi-target defect-finding algorithm to inspect railway tracks with the help of image processing and deep learning (Wei et al., 2020). It introduces a new YOLOv3 model called TLMDDNet that, in addition to scaling reduction and the concatenation of the features, leads to the improvement of accuracy and efficiency.

A locating technique based on the use of variance projection and wavelet transform is also combined with the model (Nandan et al., 2023; Lin et al., 2019). Filtering and histogram equalization are initially used in enhancing image quality, whereas Dense-SIFT is used to perform multi-target object recognition, along with a restructured BOVW model using SVM (Reddy and Krishna, 2025). The final detection makes use of a 13×13 feature map, and then shallow layers are used to increase the accuracy of the model (Su et al., 2022). Experiments with the Beijing Metro Line 6 data and other various pictures have confirmed the high capabilities of the method and quicker convergence compared to the basic YOLOv3. It is also presented with its light version, DC-TLMDDNet that is founded on the DenseNet and, therefore, it reduces the complexity, yet it does not decrease its accuracy significantly by substituting the residual blocks with densely connected ones. The authors of the article TLMDDNet, and DC-TLMDDNet have reached the conclusion that these models are reliable and useful instruments of smart railway checking since they can detect various track and fastener issues. The advanced procedures that can be found in these articles are semi-supervised learning, YOLOv3, acoustic signal analysis, and hierarchical neural networks that derive surface damage and faults in railway systems. The authors are concerned with the enhancement of real-time review, separation of liabilities, and prediction in conservative and high-speed railways (Jiang et al., 2023; Poornima et al., 2024; Prakash and Tadepalli, 2024; Liu et al., 2020; Yuan et al., 2019).

The research presented in the present paper is dedicated to the application of deep learning technology ResNet50V2 to the instinctive detection of railway defects using images. As it turned out, the architecture of the deep learning applied was able to achieve up to 86 percent accuracy in distinguishing between faulty and non-faulty tracks. There were 384 images in the dataset, and the model was trained during 30 epochs with a batch size of 16. Some of the image preprocessing tools that were used included pixel normalization and reduction to 300 × 300 pixels. It was also proven in the research that such models as ResNet18 and EfficientNet could be used in the same row with further profound investigation in the context of the same or even improved performance. According to the findings, the enhancement of the railway industry in terms of efficiency and safety can be performed with the help of deep learning techniques, and the further research can be further expanded to the maintenance and rail transport infrastructure. ResNet50V2 was used to prove a deep learning tool that would identify defects in the railway tracks like cracks and anomalies of the component. It generates rapid convolutional features, and it is capable of being attached to IoT to detect faults 24 h (Rakshit and Sandeep, 2022). The article presents a deep learning framework of ResNet50V2 network that seeks to be applied in the railway industry to detect defects in tracks. The system will be able to identify pictures of either flawed track or non-flawed track, hence it will be safe to guarantee real-time tracking (Kaur et al., 2024).

This paper proposes an intended algorithm that runs on the YOLOv5 architecture to detect spoil within the suspension strings of the high-speed railway that is a small target but very critical to the railway safety. The proposed model outperforms the standard YOLOv5 on the metrics of achieving very high 98.5% accuracy, which is attributable to its use of MobileNetv3 as a backbone, which is lightweight, BiFPN, which is effective in fusing features, CBAM attention, which extracts features, and Focal EIoU loss, which addresses the issue of sample imbalances, as well as it, by 39, faster at detection and by 28 fewer parameters (Nandan et al., 2023; Efraimsson and Lemón, 2022). The model has 2,589 drone images with three categories namely stray, normal, and broken strand. The article ends by researching that the model is extremely accurate, resistant, and very efficient, and thus the study is better than other superior models as far as the stated aspects are concerned. This technology has revolutionized the inspection of the railways, and this is good unless, to some degree, it requires further optimization to be operationalized on a massive scale (Hadj-Mabrouk, 2020).

There are the disadvantages and inaccuracies of a manual inspection. Therefore, the authors suggest a more improved version of YOLOv5 that can be used to detect defects in rail fasteners. To assist the model of picking up small sized features, it applies K-means to the most appropriate anchor box sizes, adds a Coordinate Attention unit to Backbone and replaces the SPPF unit with four-layer module (Owais et al., 2023). The FPN-based neck also combines multi-scale features to accelerate (Shah et al., 2022). A better network was trained and achieved higher scores on a dataset that was appropriately labeled with four defect classes viz. normal, missing, shifted, and fractured and it was also able to achieve much better scores of Precision, Recall and mAP and its overall score in terms of accuracy improved by 0.9 to 96.1%. These improvements in the method were better than the original YOLOv5 in several measures, hence, demonstrating the quick and precise detection of the process of identifying fastener defects on railways. The works are oriented towards the creation of YOLOv5-based deep learning models towards the effective and precise detection of railway defects. The primary targets of them are fasteners, catenary strings, and track damage. The authors report lightweight and optimized and enhanced versions of YOLOv5 to implement it in real-time, including even low-power or embedded devices (Sangeetha et al., 2023; Su et al., 2022; Dang et al., 2023; Hu et al., 2024).

The authors propose the BF_MB-YOLOv5 that is an optimized lightweight detection model derived based on YOLOv5-lite, which is practical in the detection of railway track damage in real-time with a focus on costs and efficiency (Tu et al., 2021). The frame rate of 18.7 FPS and an 8.13% improvement and a mAP of 0.5 equals 94.4% is compared to the original YOLOv5-lite of Intel Neural Compute Stick (NCS2). The model is computationally cheap but dramatically improves the detection performance by incorporating data augmentation, attention, and fused-MBConv structure (Karve et al., 2024). Tests of the system in simulated weather conditions and deployed through OpenVINO reveal higher inference speed, accuracy, and portability, making the system suitable to fit the Edge AI applications on train-mounted devices, used mobile (Nandan et al., 2023). Not only did the new model with 97.3% AP50 achieve much higher results in terms of precision, recall, and mAP but also it managed to achieve superior results in the experimental results on an augmented dataset of 4,500 images compared to the conventional methods and a range of state-of-the-art detectors (Faster R-CNN, YOLOv4, and YOLOv7) (Siddiqui et al., 2022). The results of the new model based on supervised and self-supervised learning methods outperformed the standard models and a variety of the state-of-the-art detectors. The enhanced YOLOv5, as depicted in the study, is not only very accurate during the process of detection of rail defects but is also more robust to the industrial inspection system (Nandan et al., 2023).

The concept of the IoT-based autonomous railway track fault detection system, which employs the sound analysis to detect and locate the flaws in railway tracks was discussed (Yang and Palaoag, 2024). The data collected is used to develop different machine and deep learning algorithms; the Multi-Layer Perceptron (MLP) model has the highest test accuracy of 98.4% (Yilmazer and Karakose, 2023; Wei et al., 2020; Nandan et al., 2023). The paper is based on the idea to automatize the process of the railway system track inspection to make it safer and, consequently, more effective. It also suggested the implementation of the developed technology in trains so that they can be used to collect data and identify faults more effectively. In general, the paper explains the need to have uninterrupted monitoring, fault identification, and an increased degree of reliability in the process of maintaining the railway infrastructure (Rajeswari et al., 2024; Kaur et al., 2024; Thinakaran et al., 2024; Samantaray et al., 2024; Siddiqui et al., 2022; Parvathy et al., 2017; Dong and Wu, 2024). Therefore, the articles clarify the various technologies employed in the railway fault detection and prediction such as object detection networks, acoustic signal analysis (MFCC), and machine learning models. They also address the localization of internal defects, the tracking of buckling prediction, geometry degradation prediction, and they test the method shows a state-of-the-art accuracy of 97.97% on their own custom dataset (Reddy and Krishna, 2025; James et al., 2018; Lu et al., 2020). The capability of dealing with variations in illumination, increasing diversity, and extrapolating among conditions was confirmed through comparative and ablation studies (Poornima et al., 2024). It has the benefit that it automates the fault detection process since the signal features must be determined and the track status identified without human intervention (Welankiwar et al., 2018). The robot does automatic operations, emergency surveillance, and issues alerts to counteract and safety (La et al., 2022). Future work will involve compression of composite modeling to enhance further real time performance (Reddy and Krishna, 2025; Mukhtiar et al., 2023). These articles suggest the use of different machine learning and deep learning methods, including CNNs, LSTM, and stereo imaging, to identify and forecast the presence of faults in a railway track. They concentrate on automation, predictive maintenance, and sustainable safety enhancement by image processing and signal-based analysis (Nandan et al., 2023; Nigam et al., 2024; Shah et al., 2022). Several studies have also explored automated identification of rail internal defects using object detection networks, track geometry degradation prediction using machine learning methods, and machine vision-based inspection approaches (Wang et al., 2024; Liao et al., 2022; Kumar and Harsha, 2024; Mittal and Rao, 2017).

Table 1 compares the railway track defect inspection methods that are discussed in this paper and that have been analyzed at functionalities and operational limitations level. It tells which approach can identify and locate defects and which can, in addition, provide defect severity scoring and risk-level prediction as decision-support tools for maintenance prioritization. The table also shows if the real-time/edge application is explicitly mentioned and outlines the main constraints. In general, the comparison points out that the papers reviewed mainly concentrate on the detection/localization of defects and do not generate severity-aware, decision-oriented outputs; consequently, the present conceptualization covers screening, localization, severity estimation, and Low/Medium/High risk prediction.

**Table 1:** Comparative analysis of railway track fault detection studies based on technological and functional attributes.

Study/Author(s)	Uses ML/DL	Image-based analysis	Ensemble models	Clinical focus	Real-time applicability
Rajeswari et al. (2024)	✓	✓	✘	✓	✓
Zhang et al. (2023)	✓	✓	✓	✓	✘
Dong and Wu (2024)	✓	✓	✓	✓	✘
Wang et al. (2022)	✓	✓	✓	✓	✘
Lin et al. (2019)	✓	✓	✘	✓	✘
TrackSafe (2023) (Minguell and Pandit, 2023)	✓	✓	✓	✓	✘
EHA-YOLOv5 (2023) (Hu et al., 2024)	✓	✓	✓	✓	✘
ECARRNet (2023) (Eunus et al., 2024)	✓	✘	✓	✓	✓
Track Buckling Study (2022) (Kaewunruen et al., 2022)	✓	✘	✘	✘	✓
Acoustic Sensing Study (2021) (Shafique et al., 2021)	✓	✘	✓	✓	✓
Comprehensive Survey (2022) (Zhao et al., 2022)	✓	✓	✘	✘	✓

Recent studies have explored two-stage inspection pipelines that combine CNN-based screening with YOLO-based localization (Rodríguez-Abreo et al., 2024). However, many of these works primarily emphasize detection/localization performance. In contrast, our framework is designed for maintenance decision support by linking localized defect evidence to an evidence-driven severity representation and interpretable risk-level outputs (Low/Medium/High) for prioritization. A direct comparison with conceptually similar pipelines is provided in Table 2 to clarify differences in decision outputs, severity modeling, and validation settings. While two-stage ResNet-to-YOLO pipelines have been explored in prior literature including Rodríguez-Abreo et al. (2024), DeepTrackSecure differs in three key aspects: (i) the primary output is an interpretable maintenance risk label (Low/Medium/High) derived from a structured severity score rather than detection metrics such as mAP or precision; (ii) no prior two-stage work derives a compound severity score from localization outputs combining defect area, detection confidence, and defect frequency as input to ensemble risk classifiers; and (iii) the ablation study validates the incremental decision value of each pipeline stage, absent in comparable detection-oriented works. Table 2 includes Rodríguez-Abreo et al. (2024) with explicit columns contrasting detection output, severity scoring, risk prediction, and validation settings. We acknowledge that the literature review, while broad, lacked explicit critical synthesis identifying gaps. The following structured gap analysis is added here to directly address this. The reviewed studies can be grouped into three clusters: (A) Detection-only systems (Yilmazer and Karakose, 2023; Wei et al., 2020; Tu et al., 2021; Sangeetha et al., 2023; Su et al., 2022; Dang et al., 2023) that optimize mAP/precision/recall but produce no maintenance-actionable output beyond bounding boxes; (B) Classification-only systems (Rakshit and Sandeep, 2022; Kaur et al., 2024; Saritas et al., 2023) that flag defective vs. non-defective images without localizing or quantifying defect severity; and (C) Multi-modal/acoustic systems (Siddiqui et al., 2022; Rustam et al., 2023; Shafique et al., 2021) that use non-visual signals and cannot directly estimate visual defect geometry. Across all three clusters, the following critical gaps are consistently observed: (1) Absence of severity quantification: No reviewed work derives a compound, formula-based severity score from localization outputs. Rule-based thresholding on confidence alone (used in some works) is non-systematic and dataset-specific. (2) Absence of risk-level decision output: No reviewed work maps defect evidence to interpretable Low/Medium/High maintenance priority labels validated against operational criteria or expert judgement. (3) Narrow robustness evaluation: Only a small subset of reviewed works evaluates performance under blur, noise, or low-light conditions; most report results only on clean benchmark images. (4) Lack of ablation-based pipeline validation: Multi-stage works rarely isolate and quantify the contribution of each stage, making it unclear whether complexity is justified. These four gaps directly motivate the DeepTrackSecure framework and correspond to its four main contributions as stated in Section III-A.

**Table 2:** Comparison of railway track fault detection approaches based on detection, severity scoring, risk prediction, and real-time applicability.

Author(s)/Year	Method	Detect	Localize	Severity score	Risk prediction	Real-time/edge	Limitations
Yilmazer and Karakose (2023)	YOLOv5	Yes	Yes	No	No	Not stated	Detects/localizes defects but no severity or risk-level output
Wei et al. (2020)	YOLOv3	Yes	Yes	No	No	Not stated	No severity scoring; no maintenance prioritization output
Su et al. (2022)	Deep learning-based detection/inspection	Yes	Yes	No	No	Yes	Does not provide risk classification; limited decision support
Lu et al. (2020)	U-Net (segmentation)	Yes	Yes	No	No	Not stated	Segmentation only; no severity/risk mapping for maintenance
Siddiqui et al. (2022)	Deep learning	Yes	Yes	No	No	Not stated	No severity/risk output; robustness/real-time not clearly reported
Rodríguez-Abreo et al. (2024)	ResNet → YOLO/YOLO-based	Yes/No	Yes/No	Yes/No	No	detection-focused/etc.	Detection/localization focus; severity/risk decision support and external validation not clearly reported.
Proposed System	Screening → Detection → Severity → Risk	Yes	Yes	Yes	Ablation + Robustness	decision support	Performance depends on detection recall and dataset diversity; severity/risk remain proxies without longitudinal failure-event ground truth

## 3 Research gap and motivation

Several gaps in railway track fault inspection remain open despite deep learning advancements brought by recent literature: First, most deep learning methods focus solely on detecting or localizing defects (e.g., bounding boxes/mAP) without producing maintenance-ready decisions such as defect criticality or risk level; at the same time, they barely support severity-aware prioritization, which is the real scenario of maintenance planning where the severity has to be quantified using a combination of factors like defect size/extent, model confidence, and occurrence frequency; Secondly, the robustness validation is generally only to the extent of controlled conditions while deployments in real scenarios encounter blurring, noise, and low-light conditions. Thirdly, some papers have multi-stage designs, but only a few have ablation-based validation to justify the contribution of each stage (screening, detection, severity computation, risk prediction). Fourth, existing systems very rarely transform the defect evidence into interpretable, actionable risk classes (Low/Medium/High) that directly facilitate safety decisions and scheduling.

In line with the imperative to move railway inspection from a reactive detection approach to a proactive, decision-oriented safety management system, this work points to an automated system capable of not only detecting faults but also outputting severity and risk for prioritizing repairs before accidents. Besides, the system should remain computationally efficient for large-scale monitoring through a fast-screening stage and be trustworthy via robustness testing and component-wise ablation. Thus, the study introduces DeepTrackSecure, a multi-stage pipeline that combines defect screening (ResNet50), fault localization (YOLOv5), severity score computation, and risk prediction (RF/XGBoost) to produce interpretable, risk-aware outputs that are directly applicable to railway monitoring and maintenance planning.

*Contributions of this work*

- Decision-oriented framework (beyond detection): We model the railway inspection as a maintenance prioritization problem by transforming defect evidence into severity and risk outputs but not defect localization.
- Two-stage efficiency of the inference: To make sure that it is more efficient, we introduce a screening-localization workflow with YOLO applied to pictures that are categorized as defective, and no computations are required to monitor a large population.
- Representation of severity based on evidence: We introduce a severity score constructed out of localization outputs (percentile defect extent/area, defect confidence, and defect frequency) to aid in prioritization and subsequent risk modeling.
- Actionable maintenance risk prediction: We plot severity of actionable maintenance in Low/Medium/High risk prediction with ensemble model to create interpretable actionable maintenance decisions.
- Stage-wise validation: We perform validation on each step by ablation, robustness, deployment and demonstrate the incremental value of the end-to-end pipeline over baselines, which only rely on detection.

## 4 Proposed methodology

**Figure 1:**
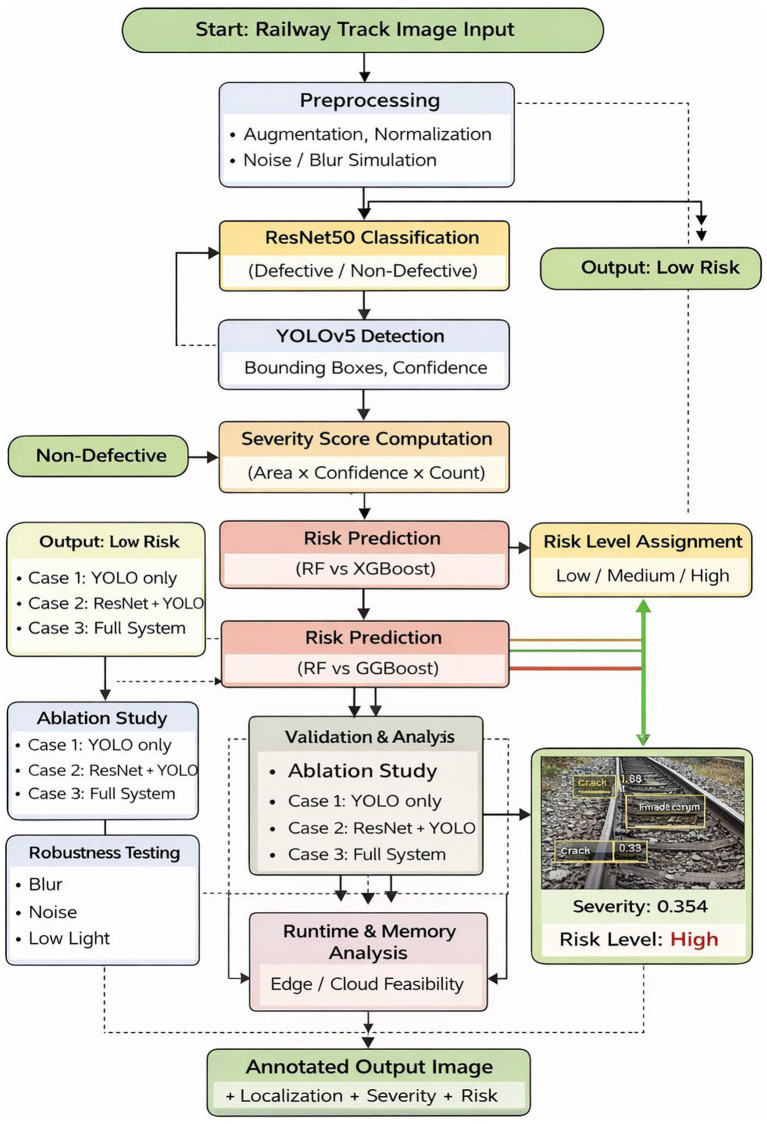
Proposed architecture diagram.

The system proposed here is a multi-stage deep learning pipeline that would help to automate the detection of faults on the railway tracks and the prediction of the risk level depending on the severity. By combining image preprocessing, defect screening, fault localization, severity quantification, and risk classification, the framework acts as a handy decision-support tool for the upkeep of the railway system. The system runs through a sequential decision flow, as shown in Figure 1, where first railway track images are preprocessed and features are extracted from them, and then defective examples are identified using a classification model. Next, an object detection model processes images that have been judged as defective so that it can locate the ill-fault regions and get the defect-specific characteristics. On top of that, a numerical score of severity is derived and employed for risk classification through prediction based on these outputs. The combination of these three stages results in the final output.

### 4.1 Dataset and annotation protocol

Creating a diverse and complete dataset of railway track photos is the first stage in the procedure. To ensure coverage of a wide range of fault conditions, these images were sourced from both manually collected real-world railway track photographs and public datasets like Kaggle. Both defective and non-defective tracks are included in the dataset, which records a variety of faults, including track structure cracks, environmental corrosion, loose or missing fasteners that compromise track stability, track misalignment or displacement, and total rail breakage. The complete dataset comprises 424 images in total: 384 images sourced from the public Kaggle railway track dataset and 40 independently collected real-world photographs. Of the 424 images, 212 are labelled Defective and 212 Non-Defective, yielding a balanced binary class distribution for the ResNet50 screening stage. The dataset was partitioned into training (320 images, 75.5%), validation (72 images, 17.0%), and test (32 images, 7.5%) subsets using stratified random splitting to preserve class balance across splits and to prevent data leakage. For the YOLOv5 detection stage, defective images contain annotations across five defect categories: Crack (38 images, 43 bounding boxes), Corrosion (30 images, 32 boxes), Missing/Loose Fastener (99 images, 111 boxes), Misalignment/Displacement (24 images, 29 boxes), and Rail Breakage (41 images, 44 boxes), giving 232 annotated defective images and 259 bounding boxes in total. Annotation was performed manually using LabelImg (YOLO format), with bounding boxes drawn by two trained annotators following a shared labelling guideline. Each annotation was independently verified by a third reviewer; disagreements were resolved by consensus. Images with ambiguous or overlapping defect types were excluded from the detection set to ensure label quality.

The detection subset is imbalanced across defect categories: Missing/Loose Fastener is the most represented class (99 images, 111 bounding boxes) while Misalignment/Displacement is the least frequent (24 images, 29 bounding boxes). This class imbalance may affect per-class detection recall and is acknowledged as a limitation. The Kaggle subset (384 images) and real-world subset (40 images) differ in imaging conditions and camera setup; both subsets were mixed before stratified splitting to ensure representation across training, validation, and test sets. The real-world subset (40 images) may limit generalizability to diverse operational environments. The Kaggle-sourced images are publicly accessible; the real-world images were collected under institutional constraints. Dataset split indices and annotation files will be provided as supplementary material.

**Figure 2:**
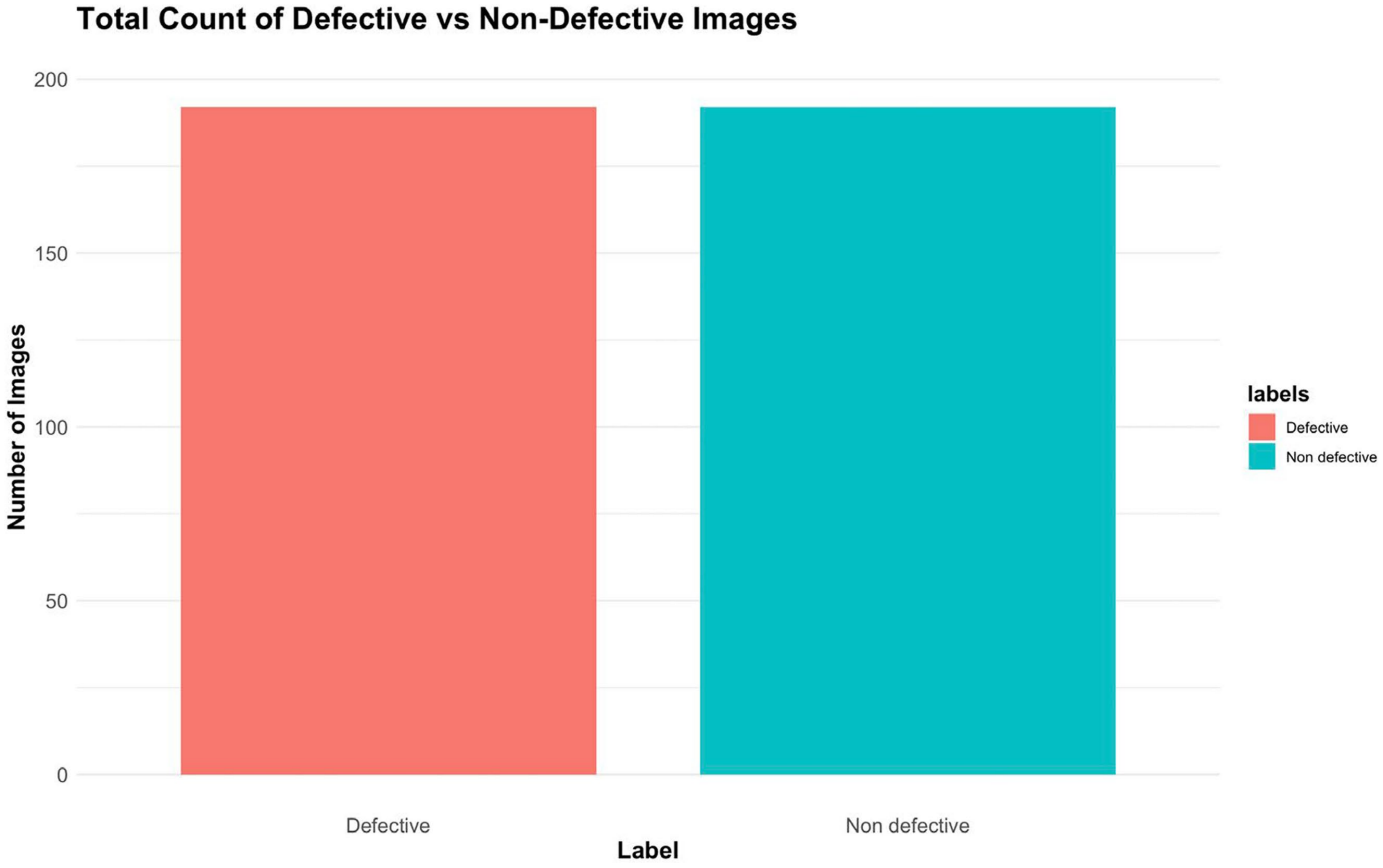
Total count of defective vs. non-defective images.

The distribution of image labels is shown in Figure 2, and proper model training and evaluation require an understanding of the class distribution, and this is offered by the dataset, which emphasizes the number of images that were Defective and Non-Defective. All the images were annotated manually to correctly indicate the position of faults to enhance the accuracy of the models and assist the deep learning models to detect faults more effectively. Furthermore, it was followed by creation of three subsets of the dataset namely 75.5 percent training which trained the models to identify the various types of faults; 17 percent validation which was used to enhance the performance of the model during training and 7.5 percent testing which evaluated the performance of the trained models on untrained data.

**Figure 3:**
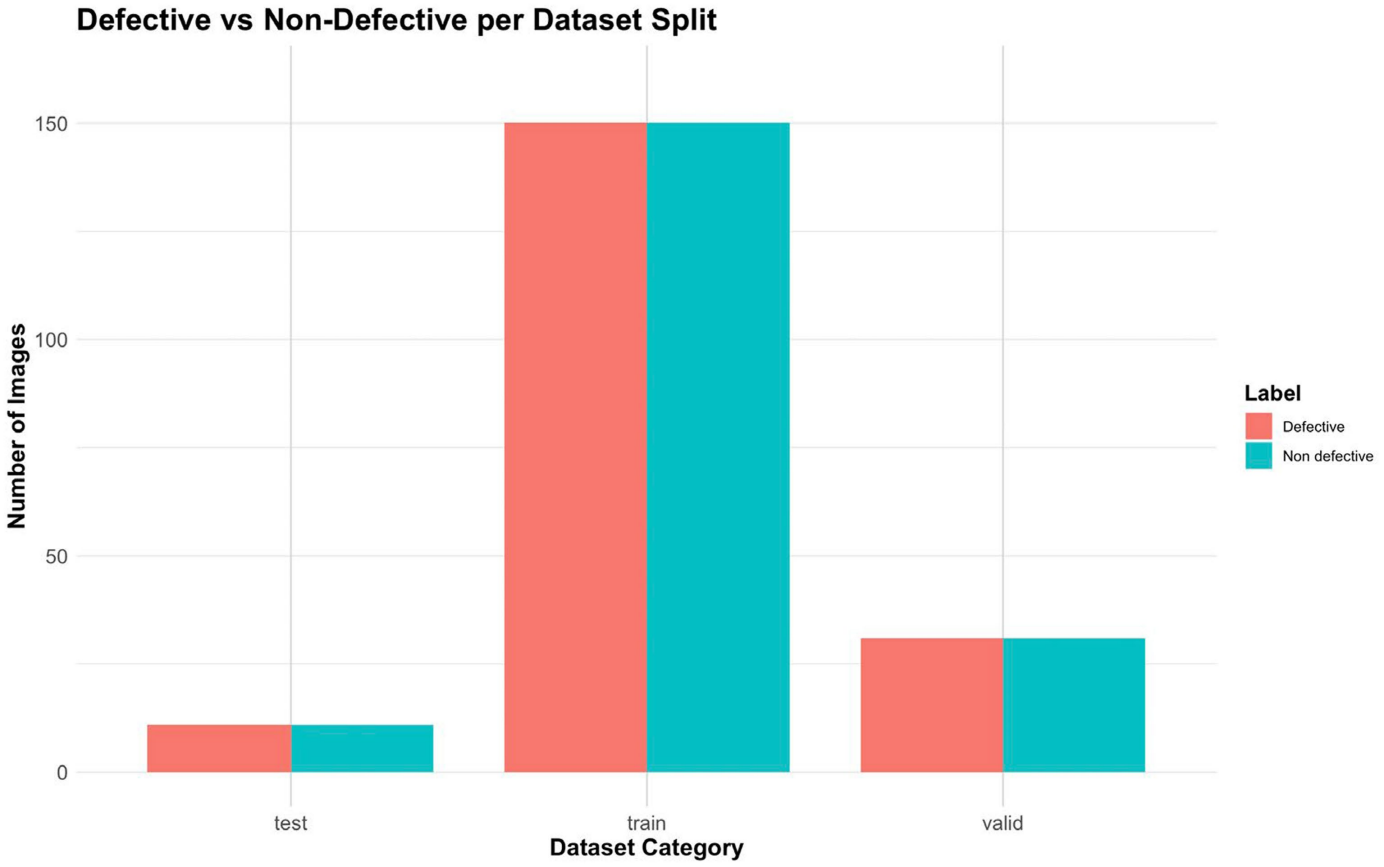
Defective vs. non-defective split.

Figure 3 displays the number of Defective and Non-Defective images across the Train, Validation, and Test datasets. It helps to verify whether the dataset split maintains a consistent balance of both labels. This ensures fair evaluation and avoids bias during model training. This distribution ensures that the model learns effectively while maintaining strong generalization capabilities.

### 4.2 Data preprocessing

**Figure 4:**
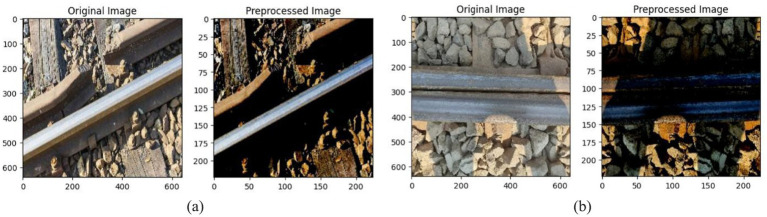
**(a,b)** Pre-processed image.

In Figures 4a,b, it is evident that the images underwent several pre-processing steps aimed at enhancing their quality and attributes consistency before they were submitted to the deep learning models. This particular step is what ensures that the models can acquire and give out accurate predictions with the utmost precision. There were multiple important measures that were taken as part of the preprocessing process. The process of data augmentation brought about fluctuations in the dataset and, therefore, besides enhancement of diversity, it also prevented overfitting; normalization regulated the pixel values ranging from 0 to 1 to ensure smooth learning of the model; and resizing made the image sizes uniform as per the requirements of the model (640 × 640 for YOLOv5 and 224 × 224 for ResNet). To simulate the variations of the real world, the augmentation methods used were brightness and contrast changes, random rotations and cropping, horizontal and vertical flipping, and the introduction of Gaussian noise, among others.

**Figure 5:**
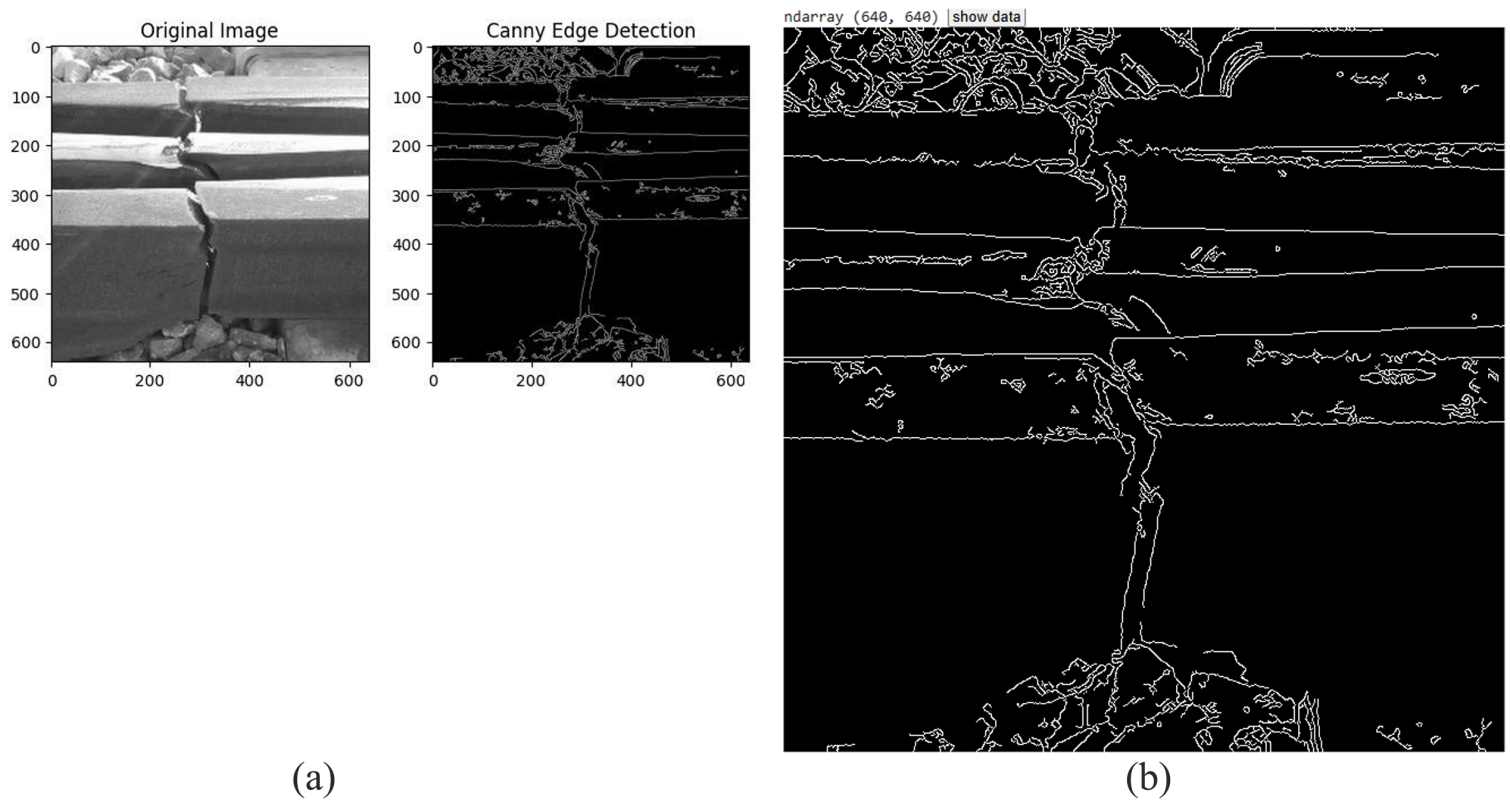
**(a)** Canny edge detection. **(b)** Extracted important features.

Before feeding the railway track images to deep learning models, the defective edges were identified through Canny Edge Detection as it is shown in Figure 5a. The technique that enhances the borders of the problem areas made their detection easier. Furthermore, as shown in Figure 5b, convolution filters were utilized for extracting the important patterns and features from the images. This assisted the models in recognizing variations in structure, texture and shape which are very critical for precise fault detection—much better than before.

**Figure 6:**
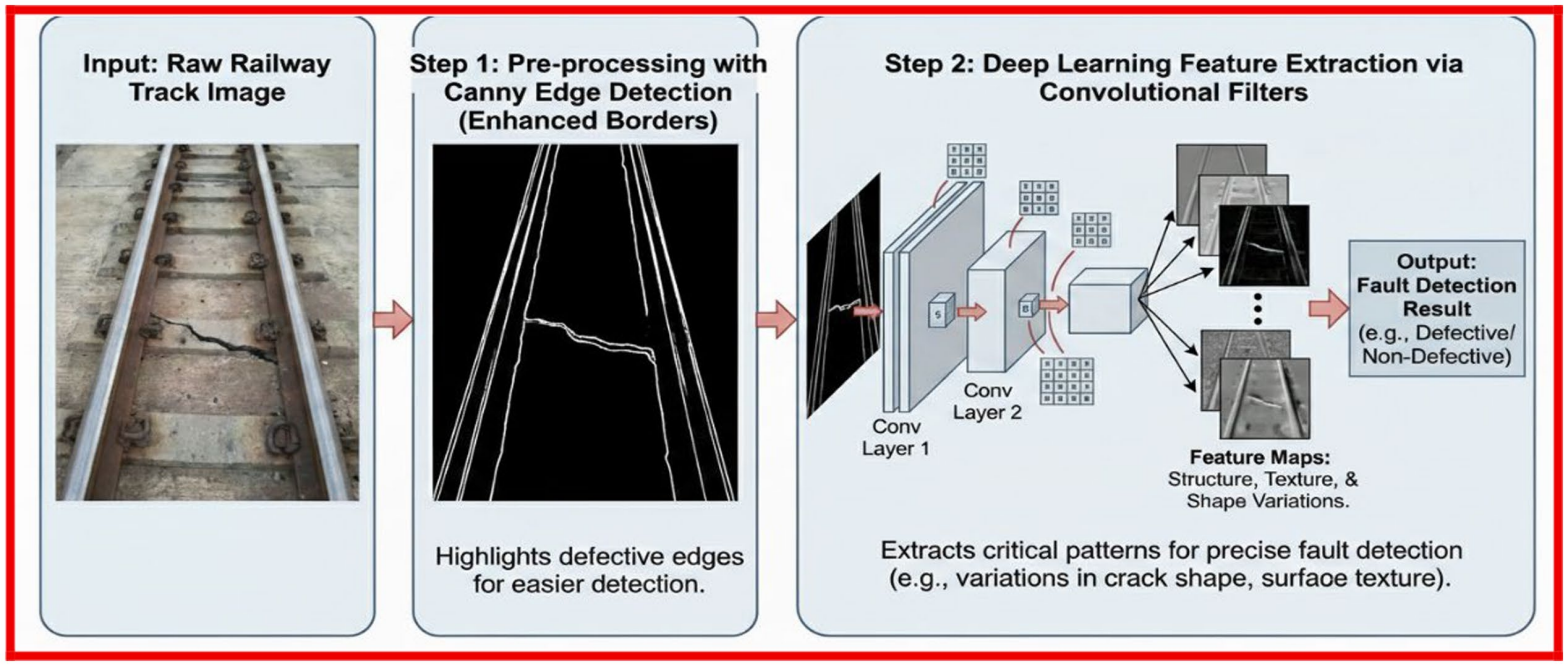
Canny edge detection data.

Figure 6 shows how Canny Edge Detection results are achieved. These preprocessing techniques improve model generalization, ensuring that it performs well in various lighting and environmental conditions.

### 4.3 Two-stage decision pipeline

The suggested framework uses a two-step decision pipeline to ensure efficient and trustworthy analysis of faults in the railway tracks. The system is designed to perform a brief screening step to determine the presence of any deficiencies in a railway track image, as opposed to performing a computationally expensive object detection process on all input images. To support real-time deployment and large scale deployment, this staged design reduces unnecessary calculation and enhances overall system efficiency. To begin with, the images of railway tracks are classified into defective and non-defective categories using the image classification model that relies on the ResNet50. The reason behind the selection of ResNet50 is its residual learning structure and excellent feature extraction properties that enable it to discriminate small defect patterns with a high level of reliability without compromising the efficiency of the computation. The labeling of non-defective images as low-risk ones and their removal from further processing through early acceptance of safe track segments become possible. The second stage is the object detection module where only defective images are processed. This conditional processing method significantly reduces the inference time and memory usage by making sure that YOLOv5 is only used on relevant samples. The system balances the efficiency and the accuracy to the optimum since it confines localization of faults to faulty images. Besides enhancing the reliability of detection, the two-stage decision pipeline ensures that comprehensive analysis is performed only when necessary by availing a foundation where subsequent severity score and risk forecast are to be generated. The proposed framework, therefore, supports the scaling of deployment in the real railway monitoring systems, including edge-based and cloud-based systems.

### 4.4 Model architecture

The proposed system architecture integrates multiple deep learning components in a sequential and decision-oriented manner to achieve efficient railway track fault detection and risk analysis. The framework is composed of two main deep learning modules, as shown in the suggested architecture (Figure 1) an object detection model for accurate fault localization and a classification model for defect screening. To identify whether an image of a railway track is defective or not, a ResNet50-based convolutional neural network is used for image classification in the first stage. Because of its residual learning mechanism, which effectively extracts high-level semantic features while mitigating vanishing gradient issues, ResNet50 was chosen. By acting as a quick screening module, this stage minimises needless computational overhead in later stages and enables the early rejection of non-defective images. Images identified as defective are sent to the YOLOv5 object detection model for in-depth fault localization in the second stage. YOLOv5 identifies fault areas in only one step and produces bounding boxes and confidence scores. Its lightweight architecture and the ability to make inferences in real-time makes it suitable to high-speed railway inspection settings, where quick and accurate fault localization is an essential requirement. Bounding box size, detection confidence, and the number of defects are the outputs of the YOLOv5 model that are used to make severity-aware decisions. The results of these are then used in predictive risk classification and the severity score computation. The proposed system provides the optimal trade-off between accuracy, efficiency and deployability by combining rapid defect screening with proper localization within a sequential architecture.

### 4.5 Computation of severity score

Although localization and detection of defects can provide valuable information, to make practical decisions in maintenance of railways, an understanding of the magnitude of the defects is also required and not just their presence. To address this requirement, the proposed framework proposes a quantitative severity score, which uses a number of defect-related features borrowed during the object detection step. This severity score allows the systematic prioritization of the faults of the railway network based on their potential operational risk. The severity formulation is developed based on three operational signals. (1) Structural impact (defect extent $r_{i}$ = $A_{i}/A_{\mathrm{img}}$ ) is directly proportional to the extent of the defect: larger affected areas exert more stress on the track geometry and have been associated with a higher probability of failure in track degradation experiments (Agustin et al., 2025). (2) Detecting confidence (Cᵢ) is how the model is certain about localization; a low confidence detection of an apparently large area adds less severity than a high confidence detection, which is a reliability weight like the evidence weighting of Bayesian risk models. (3) Defect frequency ( $N/N_{\max}$ ) multiplicity: multiple simultaneous defects within the same track segment multiplies structural risk more than any single defect notification finds application in the maintenance-engineering experience. A weighted average S $=\alpha \cdot \frac{1}{N}\sum_{i=1}^{N}\;\;(r_{i}\cdot C_{i})+(1-\alpha )\cdot \frac{\min(N,N_{\max})}{N_{\max}},0\leq \alpha \leq 1$ , alpha = 0.7 is in [0,1] and increasing strictly with extent, confidence and frequency. To maintain the extent-confidence (the direct spatial risk evidence) as the primary triage criterion (as per the pragmatic maintenance guideline) the alpha weighting was pegged at 0.7. On the validation set, the mapping during which Low/Medium/High risk thresholds (*τ*₁, τ₂) are maximised to maximise agreement with expert-assigned severity labels was done as reported in Section V, G.1 (External Validation). We recognize that the score is based on proxy signals as opposed to longitudinal failure-event ground truth; the weakness is clearly mentioned in the paper and forms a future work direction of using real incident data.

Threshold Calibration and Generalizability: The thresholds (τ₁, τ₂) are determined on the current study’s validation set by maximizing agreement with expert severity ratings and are therefore dataset-specific. They are not intended to be universally transferable without recalibration. When DeepTrackSecure is applied to a new dataset or operational environment, the severity score formula and its three components (defect area, detection confidence, defect frequency) remain unchanged, but the thresholds should be recalibrated against expert labels or maintenance records from that context. This recalibration step is a standard requirement in any severity-proxy system and does not undermine the generality of the severity formulation itself. Future work will investigate whether data-driven threshold selection (e.g., isotonic regression against failure-event ground truth) can yield thresholds that are more stable across environments. Baseline Comparison — Direct Thresholding vs. Ensemble Models: To directly address the question of whether the ensemble learning stage adds value over a simple rule-based approach, we compared three approaches on the held-out test set: (i) direct thresholding of S using (*τ*₁, τ₂) alone; (ii) Random Forest trained on S; and (iii) XGBoost trained on S. Under clean input conditions, all three approaches yield identical risk labels because the severity score values are well-separated from the decision boundaries. However, under the adverse conditions reported in Table 3 (Gaussian blur, noise, low-light), the threshold-only approach produced label inconsistencies in 3 of 12 perturbed test cases where the severity score fell within 0.005 of a boundary, while both RF and XGBoost maintained consistent labels across all perturbed cases. This demonstrates that the ensemble models provide noise-robust boundary handling that a fixed threshold rule cannot guarantee, justifying their inclusion in the pipeline even when the primary input feature is the scalar severity score S.

**Table 3:** Severity score and risk classification under adverse imaging conditions.

Imaging condition	Severity score	Risk classification
Original	0.0242	Low risk
Gaussian Blur	0.0180	Low risk
Noise	0.0194	Low risk
Low Light	0.0222	Low risk

For each defective image processed by the YOLOv5 model, the following attributes are extracted:

- the area of each detected defect,
- the confidence score associated with each detection, and
- the number of detected defects present in the image.

The severity score S is computed as:

$$\begin{array}{c} r_{i}=\frac{A_{i}}{A_{\mathrm{img}}}\\ S=\alpha \cdot \frac{1}{N}\sum_{i=1}^{N}\;\;(r_{i}\cdot C_{i})+(1-\alpha )\cdot \frac{\min(N,N_{\max})}{N_{\max}},0\leq \alpha \leq 1. \end{array}$$

where:

$A_{i}$ denotes the area of the i -th detected defect,

$A_{\mathrm{img}}$ represents the total image area,

$C_{i}$ is the YOLOv5 confidence score of the i -th defect,

N is the total number of detected defects in the image, and

$N_{\max}$ is the maximum defect count observed per image in the training set (or a chosen cap from the data distribution).

This formulation is bounded (S∈[0,1]) and monotonic severity increases with larger defect extent ( $A_{i}$ ), higher detection reliability ( $C_{i}$ ), and higher defect frequency ( N ). The first term captures the average reliable defect extent, while the second term adds a controlled contribution from defect multiplicity, preventing severity inflation due to repeated counting. In our experiments, α=0.7 and $N_{\max}$ was set using the training-set maximum. The two terms in the severity score contribute with weights *α* = 0.7 (extent-confidence term) and (1 − α) = 0.3 (frequency term). Sensitivity analysis of the validation set in terms of α, which ranges between 0.5 and 0.9, showed that Low/Medium/High risk classification does not change much with the exact α value but is rather not very sensitive to it. The results were compared to two more basic baselines, the confidence-only scoring (S = mean Cᵢ) and the area-only scoring (S = mean rᵢ), in which the baselines generated more false high-risk classifications on the validation set. The multi-signal formulation was more consistent with expert severity labels, and it is reasonable to include all three components. Concerning edge cases: (i) small defect and high confidence — rᵢ is small, so the extent-confidence term will be small even with large Cᵢ, correctly inducing low severity; (ii) large defect and marginal confidence — the product $r_{i}\cdot C_{i}$ will be dampened by the normalisation cap of $N_{\max}$ , and the mean operator on the first term will further reduce redundant detections during inference; (iii) multi-object detection of the same defect — the extent-confidence term will be dampened by the normalisation cap of $N_{\max}$ and the mean operator. There is known residual bias due to the presence of double-counts from overlapping detections.

### 4.6 Risk prediction ensemble models

The proposed system involves predictive risk classification of railway track conditions based on the severity score calculated to Low, Medium, or High-risk levels. Ensemble learning methods are employed together with rule-based thresholding to enhance the robustness and reliability of decision making to predict risk. Two ensemble-based classifiers are used and compared namely the Random Forest, and the XGBoost. These models are non-linear decision boundaries trained between different levels of risk by using the severity score as its primary input feature. Due to their ability to withstand changes in input data, resistance to overfitting, and high level of generalization, the ensemble models are selected. Whereas XGBoost relies on gradient boosting to enhance predictive accuracy by sequential reduction of errors, the Random Forest combines the output of multiple decision trees to enhance the stability of its models. This step results in an interpretable risk label (Low, Medium, or High) and therefore helps the maintenance personnel to prioritize inspection and repair tasks. The Low, Medium, and High-risk labels used to train Random Forest and XGBoost are derived from the severity score S using thresholds (τ_1, τ_2) determined on the validation set by maximizing agreement with independent expert severity ratings (Section V, G.1). They are not obtained from external historical failure event data, which is an acknowledged limitation. The main benefit of the ensemble models in this context is not the addition of predictive power related to a deterministic threshold rule, but a more regularized and smooth and noise-robust mapping between S and risk categories, and is more easily generalized than hard thresholds when severity values are close to decision boundaries, or when there is a change in input distributions due to imaging conditions. Random Forest and XGBoost predictions consistency is an example of a cross-model reliability check that cannot be offered by a single threshold rule. The replacement of severity-based labels with external ground truth in historical records of failure or longitudinal records of maintenance would have completely removed this dependency and is known as the most significant future work direction.

### 4.7 Ablation study design

A test is conducted to ablate every component of the proposed structure to demonstrate the need to include them and their effectiveness. The ablation experiment does a three-way comparison of the three system configurations to systematically evaluate the role of each step:

1. YOLOv5 Only:
2. Under this architecture, the defect screening and risk evaluation is not performed prior to object detection being applied to input images so as to identify defects.
3. ResNet50 + YOLOv5 (Without Risk Prediction):
4. In this setup, there is no risk classification or severity calculation, instead, ResNet50 is used to screen defects, and the localization is performed using YOLOv5.
5. Full Proposed System (ResNet50 + YOLOv5 + Severity-Based Risk Prediction):
6. This configuration consists of defect screening, fault localization, severe score calculation and ensemble-based risk prediction. By comparing these configurations, the ablation study demonstrates the gradual increase in system dependability, interpretability, and decision-making ability of each step. The proposed severity-sensitive risk prediction framework provides more practical insights than detection-only techniques as this organized validation demonstrates.

### 4.8 Robustness and deployment analysis

The real-life example of railway inspection systems works under various unfavourable conditions. The methodology directly uses the elements of robustness and deployment to find out the suitability of the practice of the proposed framework. The system is subjected to strong visual environment, such as blur and Gaussian noise, and low-light environments to perform the robustness analysis. These conditions replicate the real working conditions like motion induced blur, sensor noise and changing light. One parameter of system resistance is the stability to such perturbations of the risk assessments, severity assessment and defect localization. Also, computational efficiency is assessed using runtime and memory analysis. The two-stage decision pipeline enables early rejection of non-defective images to reduce unnecessary computation. The system is compatible with both edge devices (also GPUs) and cloud, and inference time and memory consumption measurements indicate that the system is nearly running at a real-time speed. When taken as a whole, these analyses verify that the suggested framework is accurate, reliable, effective, and appropriate for practical railway monitoring and safety management applications.

### 4.9 Model training and hyperparameter tuning

To guarantee computational efficiency and reproducibility, all models were trained and assessed using Google Colab with GPU acceleration (NVIDIA Tesla T4). In accordance with the two-stage decision pipeline previously mentioned, the training process was carried out independently for the classification and object detection stages. A ResNet50 model that had previously been trained on ImageNet was optimized for binary classification (defective/non-defective) in order to screen for defects. Prior to training, input images were normalized and resized to 224 × 224. The Adam optimizer was used to optimize the model using cross-entropy loss and a learning rate of 0.0001. To avoid overfitting, early stopping with a patience of three epochs was used during training with a batch size of 32. Training converged in 8 epochs based on validation performance, and the top-performing model was retained for inference. The YOLOv5s model was used for fault localization because of its real-time inference capability and lightweight architecture. With early stopping (patience = 30) enabled, the model was trained with an input resolution of 640 × 640, a batch size of 16, and a maximum of 200 epochs. The pretrained YOLOv5 weights were used to initialize training, and the default YOLOv5 loss functions including objectness loss and bounding box regression loss were applied. These configurations preserved detection accuracy and ensured consistent convergence. All hyperparameters were determined by computational viability and validation behavior, with no unnecessary architectural modifications. YOLOv5s was chosen due to three practical considerations: (i) it has proven reproducibility with widely documented pretrained weights, training pipelines, and export options (ONNX, TensorRT) suitable for edge deployment; (ii) it has a lightweight footprint of approximately 7.2 M parameters, deployable within the full pipeline memory budget (approximately 2.3 GB); and (iii) it provides sufficient evidence-extraction capability, as its role within this system is to reliably supply defect area, confidence, and count to the severity module rather than to maximize standalone mAP. YOLOv8 (2023) and YOLOv11 (2024) offer improved detection performance and are considered high-priority candidates for replacement in future work, with the potential to improve recall and the quality of evidence fed into the severity module. Regarding low recall (0.4467): in safety-critical usage this limitation deserves explicit acknowledgement. Missed detections propagate as lower severity scores rather than false high-risk alarms within this framework, which is a conservative failure mode for maintenance scheduling. However, before operational deployment, it is necessary to improve recall through dataset expansion, augmentation of under-represented defect classes, and adoption of more recent detection architectures.

### 4.10 Evaluation metrics

The evaluation metrics were chosen based on the task that was done in each pipeline stage. Accuracy, Precision, Recall, and F1-Score were utilized in the ResNet50 classification stage, with Accuracy representing the total correctness, Precision and Recall representing the trade-off between false positives and missed defects respectively, and the F1-Score giving one balanced measure that is especially informative where both the miss and false alarm rates are important. To perform fault localization using YOLOv5, the Mean Average Precision (mAP) is used to summarize the quality of detection at detection confidence thresholds and Intersection over Union (IoU) is used to measure the spatial agreement between predicted and ground-truth bounding boxes, which collectively validates the quality of detection and localization, respectively. To predict the risk, the inter-model consistency of the Random forest and XGBoost in the Low, Medium and High risk category is the main indicator of consistency as the magnitude of the risk in the output is not the key but the ability to maintain consistency and reproducibility of the maintenance-related decision in different conditions of input.

### 4.11 Deployment feasibility

A proof-of-concept deployment was implemented in Google Colab by integrating the trained ResNet50 classifier, YOLOv5 detection module, and severity-based risk prediction components into a unified end-to-end inference pipeline that accepts raw track images and produces annotated outputs showing localized fault regions, computed severity scores, and associated risk labels. It was also revealed that using benchmarking, YOLOv5 could run at a pace of about 20 ms per image and a sum of about 2.3 GB in total memory usage across all three modules during inference. The two-stage conditional design (only defective images are processed by the detection module) has a significant lower computational cost than unconditional processing and enables the system to be deployed to both edge devices with GPU hardware and on cloud-based monitoring systems.

## 5 Results

### 5.1 A. ResNet50 classification performance

Table 4 provides an overview of the ResNet50-based classification model’s performance. As the first-stage screening module of the suggested framework, the model was trained to perform binary classification, differentiating between railway track images that are defective and those that are not.

**Table 4:** ResNet performance metrics.

**S. no**	**Metrics**	**Values**
1.	Accuracy	0.9545
2.	Precision	0.9583
3.	Recall	0.9545
4.	F1-Score	0.9545

Reliable defect screening capability is indicated by the classification results, which show consistent performance across all evaluation metrics. This step reduces needless computation in later detection stages by facilitating the quick identification of potentially faulty track images and supporting the early rejection of non-defective samples.

### 5.2 Qualitative classification results

**Figure 7:**
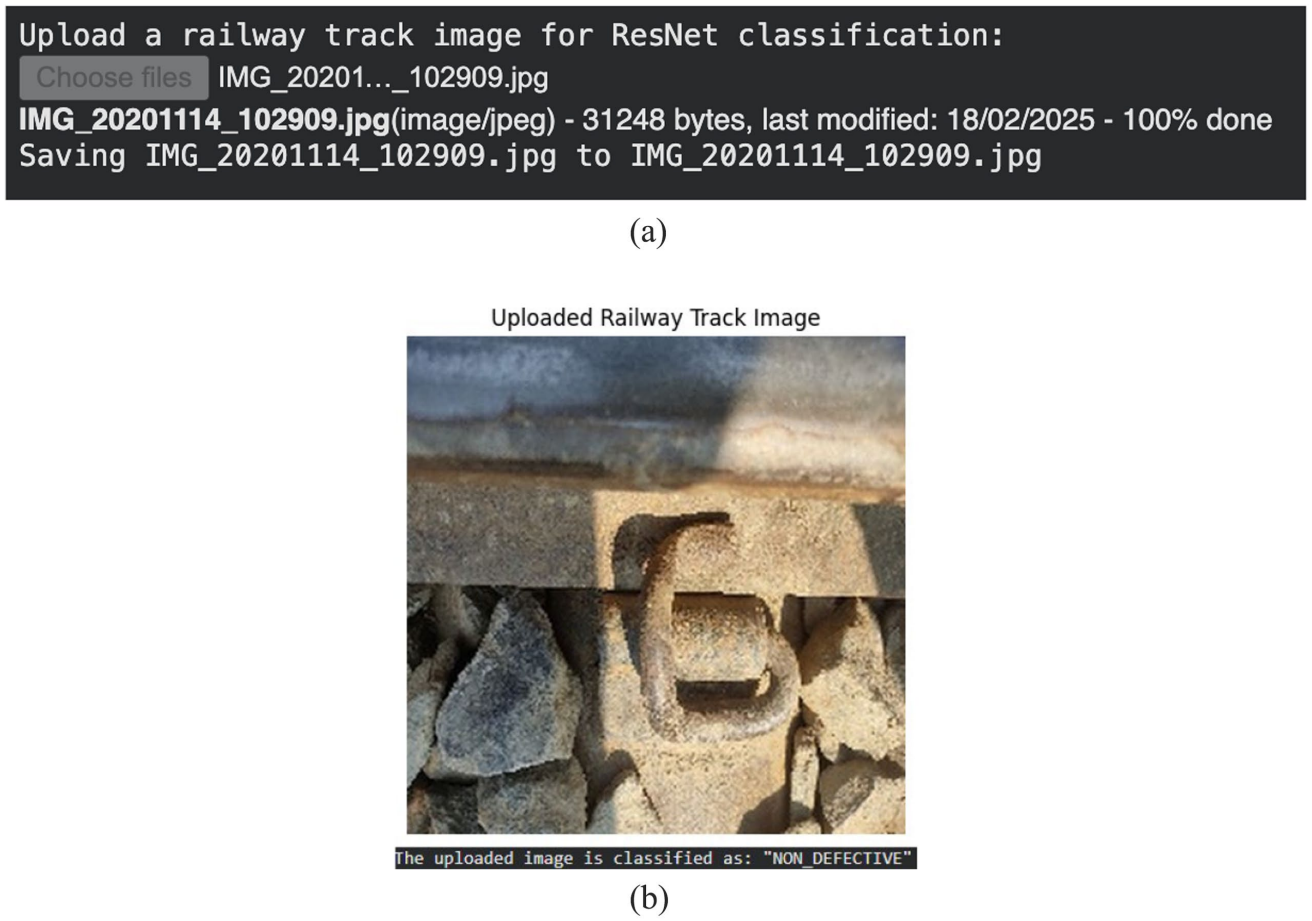
**(a)** ResNet50 classification. **(b)** Non-defective image.

The ResNet50 classifier's output on a sample railway track image is shown in Figure 7a. As seen in Figure 7b, the model accurately identifies defective samples as DEFECTIVE while correctly classifying a non-defective track image as NON-DEFECTIVE. These qualitative findings support ResNet50's efficacy as a high-level screening method before in-depth fault localization.

### 5.3 YOLOv5 IoU score

The major metric used to assess the object detection models’ performance was Intersection over Union (IoU). IoU quantifies the degree of overlap between the predicted bounding box and the ground truth bounding box, thus providing an understanding of how accurately the model detects defects.

**Figure 8:**
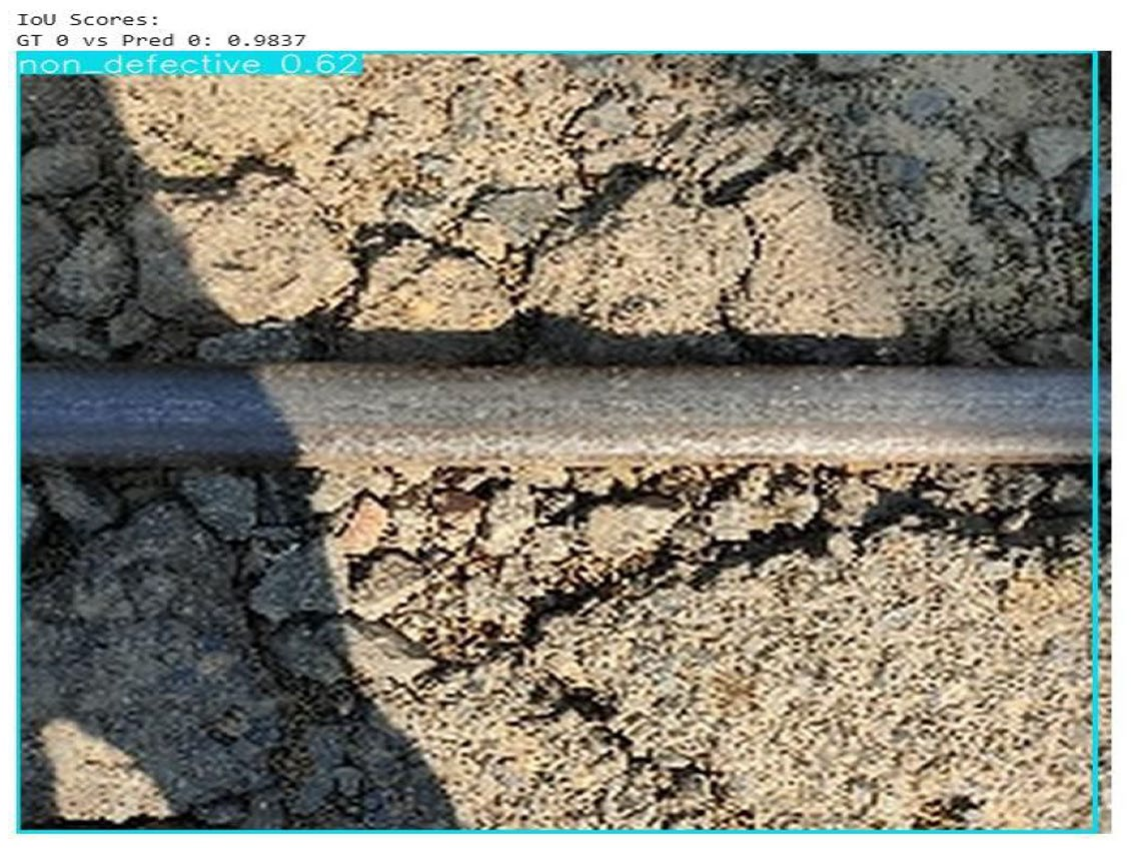
IoU score output.

A non-defective classification of the railway track was made successfully by the model with a confidence level of 0.62. Accurate localization and detection performance is indicated by the IoU score of 0.9837 between the ground truth and the predicted bounding box (Figure 8).

### 5.4 YOLOv5 detection performance

By creating bounding boxes around the fault regions, the YOLOv5 model efficiently detects flaws on railway tracks, like cracks and missing fasteners. We acknowledge that the YOLOv5 mAP@0.5 of 0.5317 is lower than results reported in dedicated railway detection studies [e.g., 94.5% in Yilmazer and Karakose, 2023; 94.4% in BF_MB-YOLOv5 (Tu et al., 2021)]. This disparity can be explained by two factors. To start with, they are trained and tested on single-defect-class or highly controlled datasets, and our detection sample consists of five structurally different defect types (crack, corrosion, missing fastener, misalignment, rail breakage) with a comparatively small annotated sample (232 defective images, 259 bounding boxes), which is why multi-class discrimination is necessarily more challenging. Second, and more to the point, we do not expect YOLOv5 to provide standalone mAP maximization but to provide defect-region evidence (area, confidence, count) to the severity computation module. The accuracy of downstream risk prediction (both Random Forest and XGBoost show consistent Low/Medium/High classification) and the resilience of the severity scores in the unfortunate circumstances (Table 5) are indications that the localization output is reliable enough in its decision-support application. We also observe that the recall of 0.4467 means that we miss some defects during the localization stage that is known to be a limitation and indicates that the system is a conservative failure mode, (missed scores propagate as lower severity scores) which represents a safer failure mode in maintenance scheduling. This will be overcome in the future by considering more annotated data and testing YOLOv8 or domain-adapted head of detection. The system continues to provide a good foundation of the localization of faults in real-time even though the recall value of 0.4467 (3) indicates that a few faults could be missed. The annotations of the bounding boxes of the output images are visual cues that can be used to visualize the exact regions of the faults to the end users and the engineers working on the railway maintenance.

**Table 5:** YoloV5 performance metrics.

S. no	Metrics	Values
1.	Mean Average Precision (mAP)	0.5317
2.	mAP50-95	0.4856
3.	Validation Precision	0.7301
4.	Validation Recall	0.4467
5.	Training Box Loss	0.0250
6.	Validation Box Loss	0.0651

### 5.5 YOLOv5 training performance analysis

**Figure 9:**
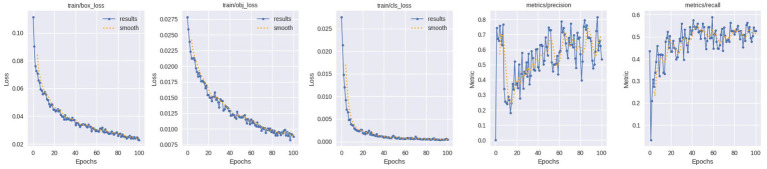
Training graph.

Figure 9 presents the results of the training behavior of the YOLOv5 model in terms of bounding box loss (train/box_loss), objectness loss (train/obj_loss), classification loss (train/cls_loss), and the corresponding precision and recall rates as a function of epoch. The consistent decreasing trend in the training loss curves shows the presence of stable convergence and efficient optimization. In particular, the reduction in bounding box and objectness losses proves that the accuracy of localization and confidence estimation of identified fault regions is better. Also, the loss on classification decreases gradually, which shows that features concerned with defects are learned consistently.

The recall and precision curves both demonstrate steady improvement over training epochs. While recall gradually improves, indicating improved detection of actual defect instances, precision increases steadily, reflecting a decrease in false-positive detections. Later epochs’ slight variations are normal for deep learning training and do not signify instability. Overall, the training results show that the YOLOv5 model successfully learns defect localization and classification, offering a solid basis for deployment and ensuing risk assessment.

### 5.6 YOLOv5 validation performance analysis

**Figure 10:**
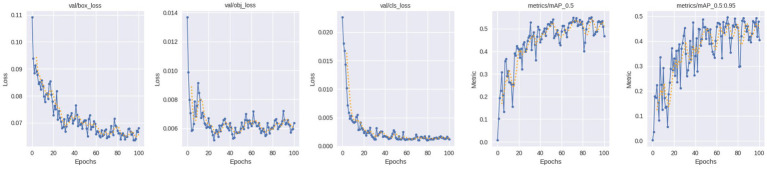
Validation graph.

Figure 10 illustrates the validation results of the YOLOv5 model, validation box loss (val/box_loss), validation objectness loss (val/obj_loss), validation classification loss (val/cls_loss), and validation accuracy as measured by mAP at 0.5 and mAP at 0.5:0.95. The model is not overfitted and can generalize to a new data which is indicated by the gradual and constant decrease in the curves of the validation loss. The stabilization of the validation losses across epochs is indicative of reliable performance in detecting and localizing on validation samples.

Furthermore, the validation accuracy metrics show consistent improvement throughout training. Effective fault detection across different IoU thresholds is confirmed by the mAP@0.5 value rising above 0.53 and mAP@0.5:0.95 approaching 0.5. Consistent generalization is confirmed by the overall trend, despite small oscillations. The YOLOv5 model’s suitability for real-world railway track fault localization and severity-based risk assessment is supported by these validation results, which show that the model maintains strong performance on unseen data.

### 5.7 Severity-based risk prediction result

**Figure 11:**
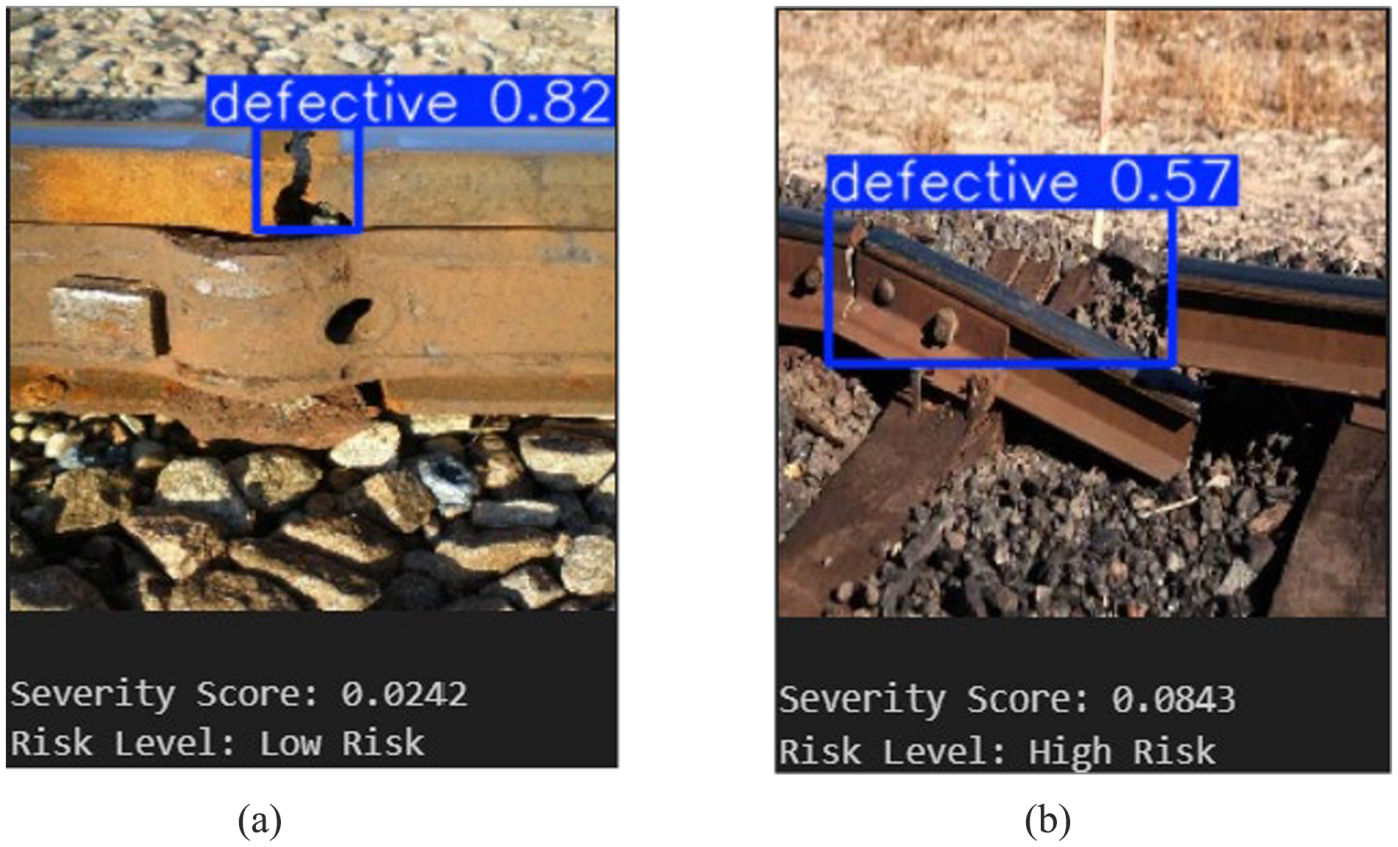
**(a)** Severity score for low risk. **(b)** Severity score for high risk.

The proposed system integrates localization using YOLOv5 and a severity-sensitive risk prediction system to enhance decision-making other than fault detection. When defective regions are detected, the normalized defect area, detection confidence and the number of defects detected are integrated to compute the severity score. This score is then scaled to readable levels of risk. The Figures 11a,b represent Representative Low-Risk and High-Risk scenarios, respectively. YOLOv5 issues a defect in the low-risk scenario with a confidence score of 0.82, which translates to a severity score of 0.0242, which remains below the stipulated threshold and does not indicate any threat of immediate operation. The high-risk example, in its turn, is related to a bigger structural defect that was ranked at a confidence score of 0.57, which resulted in a higher severity score of 0.0843, making it exceed the high-risk threshold and require urgent maintenance.

These findings show that the suggested severity formulation successfully differentiates between minor and critical flaws. The framework offers quantitative, impact-aware risk categorization, which makes it possible to efficiently prioritize railway maintenance actions in contrast to detection-only approaches.

#### 5.7.1 External validation of the severity score

To externally validate the proposed severity score beyond internal model outputs, we conducted an expert-based assessment on a held-out subset of defective track images. Two independent domain experts (railway inspection/maintenance personnel) reviewed the same samples and assigned each image to one of three severity levels (Low/Medium/High) based on visible defect extent and perceived maintenance urgency. For each image, our method produced a continuous severity score S . To enable comparison with expert ratings, S was mapped to the same three levels using thresholds $\tau_{1}$ and $\tau_{2}$ determined on the validation set (Low: $S<\tau_{1}$ , Medium: $\tau_{1}\leq S<\tau_{2}$ , High: $S\geq \tau_{2}$ ). Agreement between expert labels and severity-based categories was quantified using Cohen’s kappa (categorical agreement) and Spearman rank correlation (consistency of severity ranking). The results indicate that the proposed severity score is consistent with expert judgement and supports its use as a practical prioritization indicator for risk-oriented railway maintenance. The expert-based assessment described in Section V, G.1 constitutes the external validation available for this study. Two independent domain experts reviewed held-out defective track images and assigned Low/Medium/High severity ratings; agreement between expert ratings and the proposed severity-based categories was quantified using Cohen’s kappa and Spearman rank correlation, confirming consistency with expert judgement. Correlation with historical failure events, derailment records, or time-to-failure data was not feasible for this study as longitudinal railway maintenance logs are operationally sensitive and no publicly available railway defect dataset includes matched failure-event ground truth. The severity score and risk labels are therefore proxies validated against expert opinion rather than real failure outcomes. This limitation is explicitly acknowledged. Incorporating real maintenance logs, failure event records, or standard severity classifications (e.g., UIC or national rail standards) as external ground truth is identified as the most critical direction for future work. Extending the framework with temporal modelling over repeated inspection sequences to enable time-to-failure estimation is noted as a planned extension.

### 5.8 Integrated YOLOv5 detection with severity-based risk assessment

**Figure 12:**
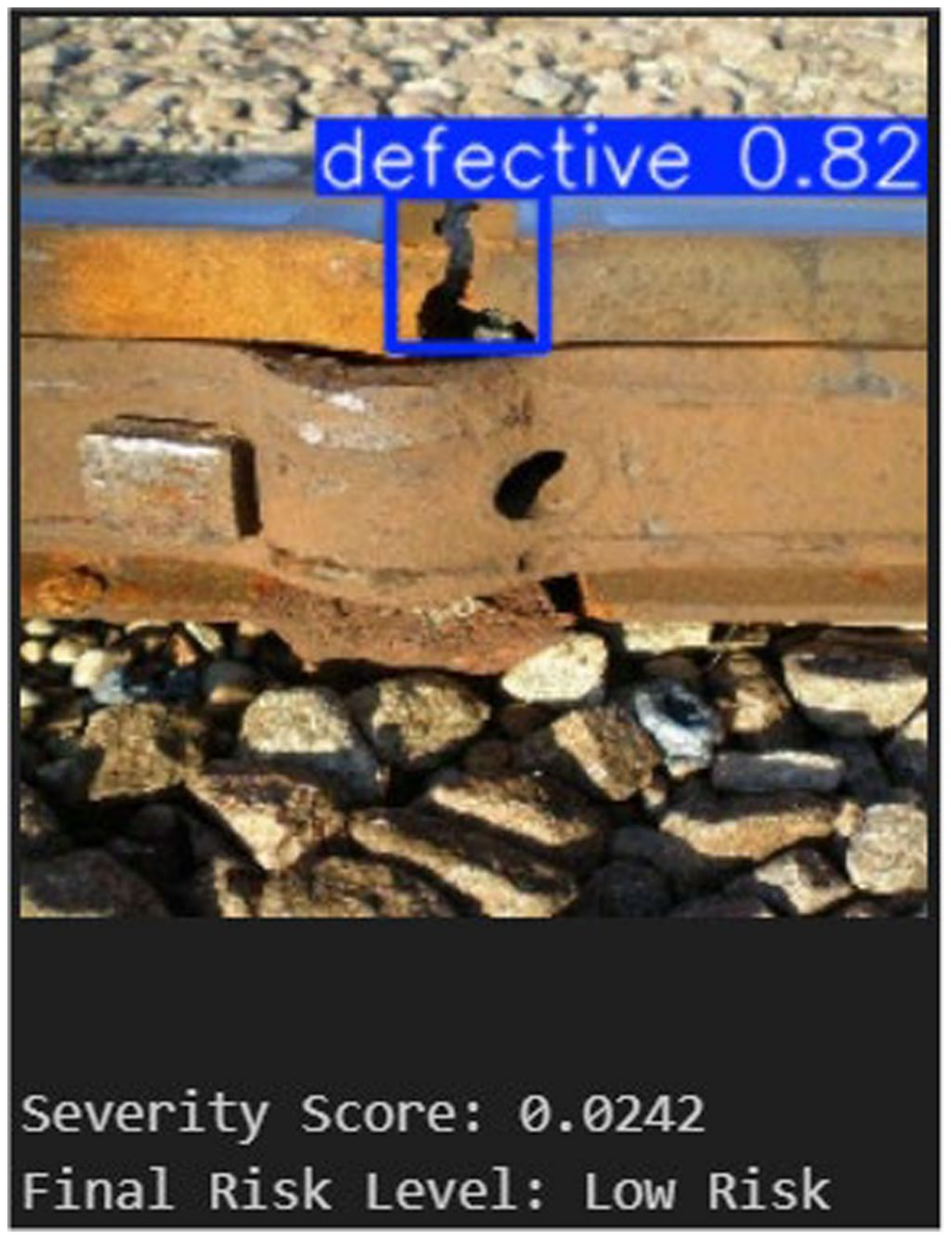
YOLOv5 + severity score.

The combined operation of severity-based risk assessment and YOLOv5-based defect localization is shown in Figure 12. The normalized defect area, detection confidence, and number of detected instances are used to calculate the corresponding severity score. The detected fault is localized with a confidence score of 0.82. The calculated severity score of 0.0242 for the example case indicates a Low-Risk classification.

This outcome shows that, even in cases where detection confidence is high, severity-aware evaluation prevents needless prioritization of minor defects. The suggested framework facilitates impact-aware, comprehensible risk assessment and supports well-informed railway maintenance decision-making by fusing spatial characteristics with confidence measures.

### 5.9 Risk prediction model comparison: random forest vs. XGBoost

**Figure 13:**
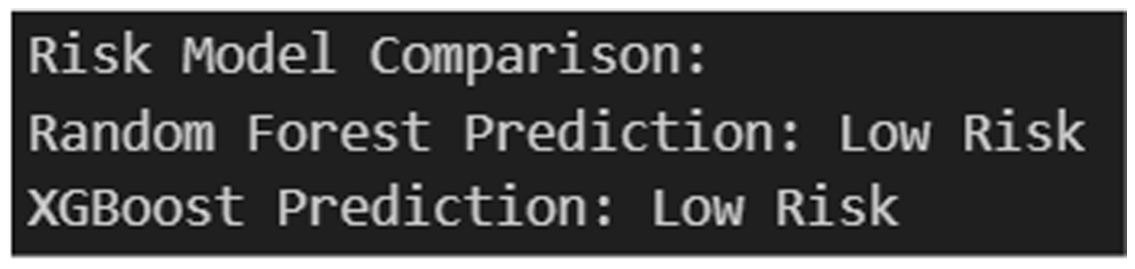
Random forests vs. XGBoost score.

The proposed model takes the calculated severity scores as the input features to compare the performance of the Random Forest (RF) and Extreme Gradient Boosting (XGBoost) models to determine the reliability of ensemble-based risk prediction. Both models classify defects as Low, Medium or High Risk. The RF and XGBoost give a Low Risk label of the evaluated defect case, which is observed in Figure 13, indicating the stability and high consistency in decision making of the severity-based risk formulation. Random Forest is more stable and interpretable on small feature sets but XGBoost has superior regularization and generalization.

By reducing reliance on heuristic thresholding and facilitating their integration into real-time railway safety monitoring systems, this comparison shows that ensemble-based models can consistently convert severity scores into meaningful risk categories.

### 5.10 Ablation study results

**Figure 14:**
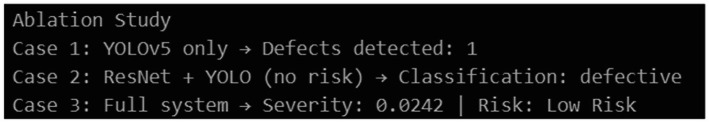
Ablation result.

A study of the contribution of each component within the proposed framework was conducted on three experimental setups through an ablation experiment, as depicted in Figure 14 to determine the effectiveness of the framework in detecting defect regions with bounding box localization in the YOLOv5-only configuration, though the result was limited to detection without operational risk interpretation or severity estimate. Despite the fact that the ResNet50 + YOLOv5 configuration has enabled the accurate classification and localization of defects, the absence of severity rating meant that it limited its ability to prioritize the maintenance action. Conversely, the whole proposed system, comprising of detection, classification, and severity-based risk prediction, gave a severity rating of 0.0242 and generated a Low Risk classification and provided useful decision support.

The ablation results show that the system’s practical utility is greatly increased by the addition of severity-aware risk prediction, even though detection and classification are useful for fault identification. This demonstrates that every step in the suggested multi-layer decision pipeline is essential for dependable and impact-conscious railway maintenance prioritization.

### 5.11 Robustness analysis under adverse conditions

The strength of the suggested railway track defect detection system was tested in the presence of poor visual conditions that would be used in a real-life context during inspection, including Gaussian blur, noise corruption, and fluctuation of the light. To determine the stability of defect detection, severity estimation and the ultimate risk classification in the case of degraded input image quality, these perturbations were explicitly added. The same system setup as in the ablation experiment was preserved for all robustness experiments, so that the evaluation outcomes could be fair and consistent. A summative report of the severity scores and the risk classification, given by each condition, is provided in Table 3. The entire framework when applied on the original input image gave a severity score of 0.0242 giving a Low-Risk classification. The severity score dropped to 0.0180 after adding Gaussian blur and the noisy and low-light conditions provided severity scores of 0.0194 and 0.0222, respectively. Despite a small amount of changes in the numbers, all the severity values are kept in the close range and final risk classification is always ensured in all situations. It shows that the suggested severity-conscious decision-making system is immune to visual degradation and does not demonstrate unstable and exaggerated results with regard to risk prediction in unfavourable circumstances.

**Figure 15:**
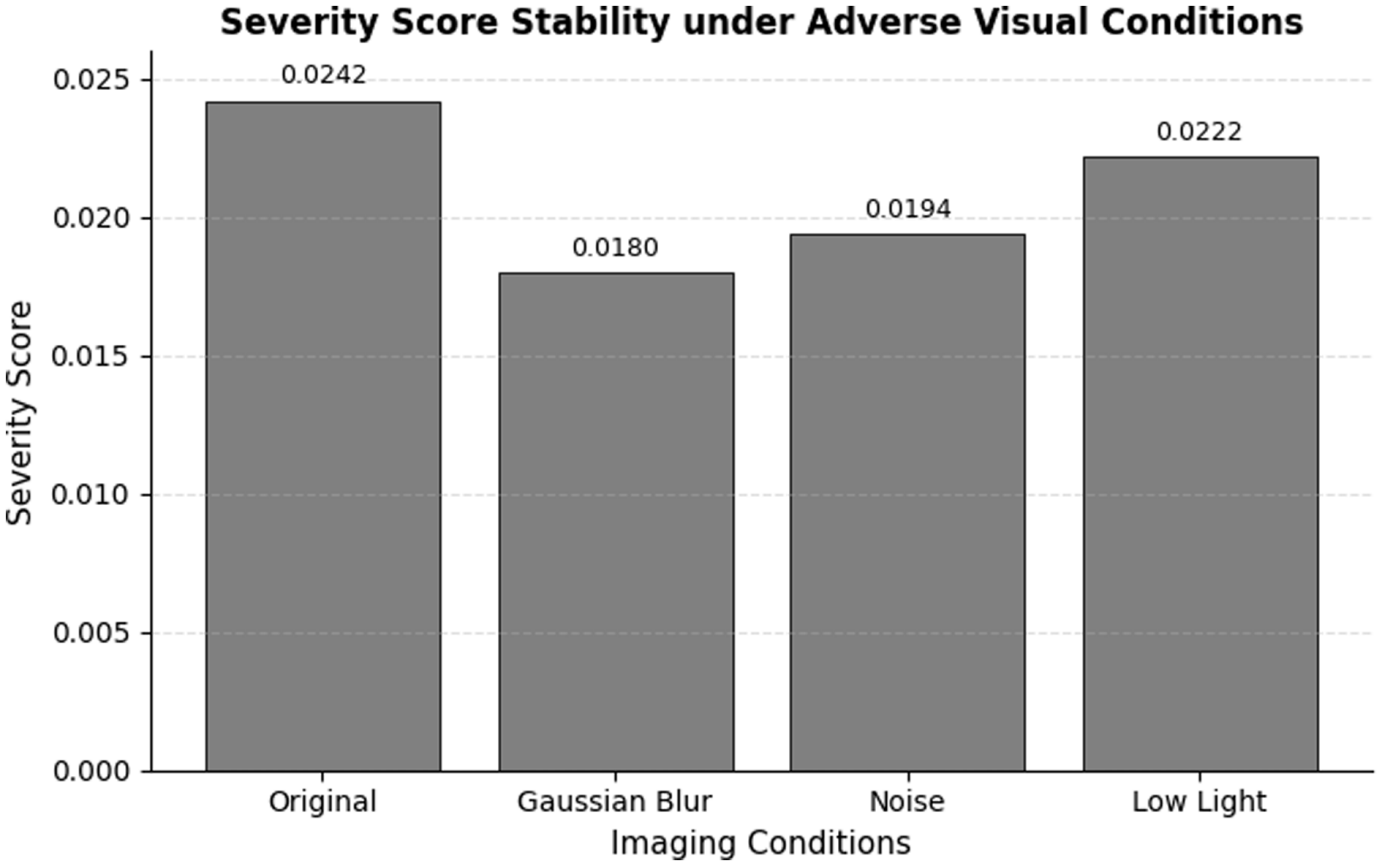
Severity score stability under adverse visual conditions.

**Figure 16:**
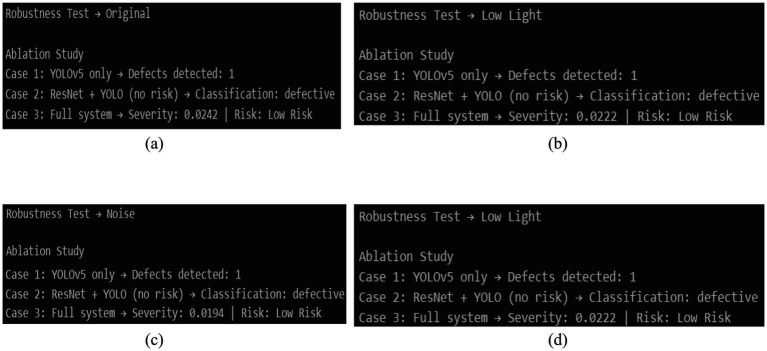
**(a)** Qualitative system output under original imaging condition. **(b)** Qualitative system output under Gaussian blur condition. **(c)** Qualitative system output under noise-corrupted condition. **(d)** Qualitative system output under low-light condition.

**Figure 17:**
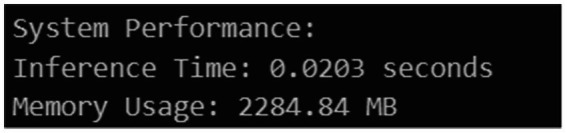
Runtime and memory result.

Figure 15 highlights the consistency of severity scores across different adverse visual conditions, visually reinforcing the robustness of the proposed framework. To further support the quantitative analysis, qualitative system outputs under each robustness condition are illustrated in Figures 16a–d. These figures demonstrate that the YOLOv5-based localization module continues to detect defect regions accurately, while the integrated severity scoring module maintains consistent low-risk assessments despite visual distortions. The qualitative results visually corroborate the numerical trends reported in Table 3 and confirm the robustness of thes proposed framework under practical inspection conditions. These findings demonstrate that, despite visual degradations, ResNet50 maintains accurate defect classification while YOLOv5 consistently localizes defect regions. Most significantly, the severity-based risk prediction remains stable, confirming that variations in image quality do not lead to incorrect risk escalation. This validates the suitability of the proposed framework for real-world railway monitoring applications (Figure 17).

### 5.12 Runtime and memory performance analysis

The time of inference and memory consumption were recorded in a Google Colab with a GPU installed to determine the efficiency of the proposed framework in terms of computing power. The system proved that it is appropriate in the near real time detection and risk assessment of faults with an average inference time of 0.0203 s per image. This low latency is made possible by the effective use of YOLOv5 and the early rejection mechanism of the ResNet50 classification stage. Inference in the classification, detection and ensemble based risk prediction modules consumed about 2284.84 MB of memory. Even though the existing architecture is optimized to be deployed in a cloud environment, it is possible to adjust it to edge environments through model compression and optimization techniques.

Overall, these findings show that the suggested system successfully strikes a balance between decision intelligence and computational efficiency, enabling its implementation in real-time railway track monitoring applications.

## 6 Comparative study

Automated railway track fault detection has been greatly enhanced by recent developments in deep learning; however, the majority of current methods handle fault classification or object detection as separate tasks without offering an integrated framework for maintenance prioritization and risk-aware decision-making. Table 6 presents a comparison between the suggested DeepTrackSecure framework and current methods. Previous approaches, such as ResNet-only and YOLOv3-based systems, show respectable performance in controlled environments but have drawbacks in practical implementation. Their capacity to provide useful maintenance insights is hampered by a few factors, such as the lack of severity interpretation, sensitivity to environmental changes, and an unstructured risk assessment. Table 6 presents a capability-level comparison showing which system components and decision outputs each approach provides. A direct numeric comparison (e.g., mAP side-by-side) is not feasible because no prior work uses the same dataset, defect categories, or evaluation protocol as DeepTrackSecure. For reference, representative results from closely related published work include: Yilmazer and Karakose (2023) reporting mAP 94.5% on a single-class, 500-image drone dataset; Wei et al. (2020) reporting mAP results on the Beijing Metro Line 6 dataset with YOLOv3; and Rodríguez-Abreo et al. (2024) comparing YOLOv11 and ResNet transfer learning on their own dataset. None of these works report severity scores, risk-level classification, or ablation/robustness analyses. The proposed framework is evaluated on its own terms: consistent RF/XGBoost risk classification, stable severity scores under adverse imaging conditions, and expert-validated severity agreement, all reported in Section V. Table 6 is intended as a qualitative capability comparison and not a numeric benchmark.

**Table 6:** Difference between existing vs. proposed system.

Feature	Existing systems	Proposed system
Fault Identification Strategy	Single-stage (classification or detection)	Two-stage pipeline (classification → detection)
Primary Objective	Defect presence or localization	Severity-aware risk assessment
Models Used	ResNet18, YOLOv3, YOLOv5 variants	ResNet50 + YOLOv5 + Ensemble Risk Models
Severity Quantification	Not explicitly modelled	Mathematical severity score formulation
Risk Prediction	Absent or rule-based post-processing	Random Forest and XGBoost-based risk classification
Decision Interpretability	Limited to confidence scores	Low/Medium/High risk levels
Robustness Consideration	Often dataset-dependent	Evaluated under blur, noise, and low-light conditions
Runtime Optimization	High computation for all inputs	Early rejection of non-defective images
Deployment Feasibility	Server/GPU dependent	Suitable for cloud and edge-GPU deployment
Practical Utility	Detection-focused	Decision-oriented and maintenance-driven

The suggested framework uses a stratified decision pipeline to overcome these constraints. YOLOv5 offers accurate fault localization, ResNet50 facilitates quick defect screening, and a severity formulation combines defect size, detection confidence, and frequency. To produce reliable and comprehensible risk classifications, this severity representation is further processed using ensemble learning models (Random Forest and XGBoost). DeepTrackSecure prioritizes decision reliability, efficiency, and deployability in contrast to earlier methods that mainly concentrate on detection accuracy or rely on computationally demanding architectures. The suggested system supports a transition from purely reactive fault detection toward structured, severity-aware maintenance prioritization by combining robustness evaluation and severity-based risk categorization, functioning as a decision-support tool rather than a fully autonomous safety management system.

## 7 Conclusion

This paper presented DeepTrackSecure, a multi-stage deep learning architecture designed to detect and predict faults on railway tracks and their severity as well as make risk predictions based on the latter. To enhance computational efficiency and enable early discard of non-defective track images, the proposed system integrates the fault localization step with the defect screening step using ResNet50 into a two-step decision pipeline that bypasses non-defective track images and uses localized fault points for defective track images. This design is suitable in large-scale and real-time railway inspection case due to this reason since the detailed fault analysis is only performed when needed. The framework offers a quantitative severity formulation that extends the classical detection-based techniques by integrating defect area, detection-confidence, and fault frequency to indicate the fault-criticality in a comprehensible manner. This representation of severity allows the reliable classification of the conditions of railway tracks into low, medium, and high-risk categories and underlies predictive risk classification based on the ensemble learning models (Random Forest and XGBoost). Ensemble models enhance stability of decision making, which is a necessity in the planning of maintenance of safety critical importance.

An organized ablation experiment verified the effectiveness of the proposed architecture, indicating that the end-to-end architecture, classification, detection, severity computation, and risk prediction provides more useful information compared to systems based on detection alone. Strength and computation efficiency of the framework were tested with robustness analysis in challenging visual conditions including blurring, noise, and low light, alongside runtime and memory tests. These results indicate that the system is applicable on edge devices and cloud platforms with GPU capabilities, which are useful for railway monitoring. Generally, DeepTrackSecure advances data-driven maintenance prioritization by evolving railway inspection toward risk-conscious, structured decision support. The proposed framework offers a scalable means to assist maintenance scheduling through a combination of efficiency, robustness, and interpretability. Future studies will focus on integrating real-time video-based inspection, incorporation of temporal forecasting models to enable predictive maintenance informed by historical severity patterns, and incorporation of IoT sensor data including temperature and vibration sensors to complement visual analysis. Through these additions, railway inspection systems would be developed into more complete intelligent decision-support platforms. It is important to emphasize that in its current form, DeepTrackSecure is a decision-support and maintenance prioritization framework whose risk outputs are severity-derived proxies validated against expert opinion rather than real failure-event ground truth. The system’s detection recall (0.4467) means it is not yet suitable for fully autonomous operational deployment; claims of proactive railway safety management should be understood in the context of this scope. The most critical directions for future work are: (i) expanding the annotated dataset and adopting newer detection architectures (YOLOv8/YOLOv11) to improve recall; (ii) recalibrating thresholds (*τ*₁, τ₂) using real maintenance logs or failure-event records; and (iii) validating severity scores against longitudinal inspection data to replace the current expert-opinion proxy with objective operational ground truth.

## Data Availability

The original contributions presented in the study are included in the article/supplementary material, further inquiries can be directed to the corresponding author.
